# Frequency of weight and body composition increases in advanced non‐small cell lung cancer patients during first line therapy

**DOI:** 10.1002/jcsm.13534

**Published:** 2024-09-17

**Authors:** Philip Bonomi, Hita Moudgalya, Sandra L. Gomez, Palmi Shah, Sanjib Basu, Marta Batus, Levi B. Martinka, Ahmed Abdelkader, Iphigenia Tzameli, Sonia Cobain, Susie Collins, Edmund J. Keliher, Danna M. Breen, Roberto A. Calle, Mary Jo Fidler, Jeffrey A. Borgia

**Affiliations:** ^1^ Division of Hematology/Oncology Rush University Medical Center Chicago IL USA; ^2^ Department of Anatomy and Cell Biology Rush University Medical Center Chicago IL USA; ^3^ Department of Clinical Nutrition Rush University Medical Center Chicago IL USA; ^4^ Department of Radiology Rush University Medical Center Chicago IL USA; ^5^ Biostatistics, School of Public Health University of Illinois Chicago Chicago IL USA; ^6^ Internal Medicine Research Unit Pfizer Inc. Cambridge MA USA; ^7^ Global Biometrics and Data Management Pfizer R&D UK Ltd Sandwich UK; ^8^ Early Clinical Development Pfizer Inc. Cambridge MA USA; ^9^ Department of Pathology Rush University Medical Center Chicago IL USA

**Keywords:** body composition, body weight, cancer cachexia, chemotherapy, immunotherapy, non‐small cell lung cancer

## Abstract

**Background:**

The primary objective of this study was to assess the frequency of body composition increases and their relationships to changes in body weight in two cohorts of real world, treatment‐naïve, advanced non‐small cell lung cancer (NSCLC) patients. One cohort received the current standard of care (CSOC), which consisted of immunotherapy and newer chemotherapy regimens, and the other cohort was treated with the former standard of care (FSOC), consisting only of older platinum‐containing regimens.

**Methods:**

CSOC (*n* = 106) and FSOC (*n* = 88) cohorts of advanced NSCLC patients were included in this study. Weights were collected at each clinical visit, and body composition analysis from routine chest computed tomography via automated segmentation software assessed at baseline and at 6 and 12 weeks. Standard statistical methods were used to calculate relationships between changes in weight and in body composition.

**Results:**

The CSOC cohort contained 106 stage IV NSCLC patients treated between 16/12/2014 and 22/10/2020 while the FSOC cohort contained 88 stage III/IV NSCLC patients treated between 16/6/2006 and 18/11/2014. While each cohort exhibited decreases in median weight, body mass index (BMI), mean skeletal muscle index (SMI) and subcutaneous adipose tissue index (SATI) at the 6 and 12 week time points, a subset of patients experienced increases in these parameters. Using a threshold of ≥2.5% increase for weight, BMI, SMI, and SATI at the 12 week time point, both cohorts showed similar (20.5% and 27.3%) increases in these parameters. With a cut point of ≥5% increase at 12 weeks follow‐up, 8.0% to 25.0% of the patients gained ≥5% in weight, BMI, SMI and SATI. Comparing these results in each cohort showed no significant differences. Pearson coefficients for weight change related to changes in SMI and SATI at 6 and 12 weeks ranged from 0.31 to 0.58 with all *P* values <0.02. Pearson coefficients for weight change at 12 weeks related to changes in VATI and IMATI ranged from 0.26 to 0.47 with all *P* values <0.05. Comparison of Pearson coefficients for each cohort showed no significant differences.

**Conclusions:**

Although decreases in median weight, BMI, SMI and SATI were observed in both cohorts, similar percentage of patients in each cohort experienced increases in these parameters. These findings, plus the positive correlations between longitudinal measurements of weight, muscle mass and adipose tissue, indicate that weight gain in these patients involves increases in both muscle mass and adipose tissue. Upon validation, these findings could have implications for clinical trial design and for translational research in cancer cachexia.

## Introduction

Anorexia and weight loss occur commonly in advanced non‐small cell lung cancer (NSCLC) patients.^1^, ^2^ Confounding this situation, many patients treated with platinum‐containing chemotherapy frequently experience anorexia, nausea and vomiting as side‐effects. While it is counter‐intuitive to expect that weight gain might occur in advanced NSCLC patients during treatment with platinum‐containing chemotherapy, there are reports from retrospective, small single‐institutional studies describing such a phenomenon with concurrent chest radiation in stage III NSCLC patients.^3^, ^4^, ^5^ More recently, weight gain was also found in post hoc analyses of longitudinal weight measurements in large first line chemotherapy NSCLC clinical trials.^6^, ^7^, ^8^ Weight gain >5% was observed in 18.3% of 2301 NSCLC patients in one study^7^ and 11.7% of the patients in a smaller study (1088 patients).^6^ In another study, weight gain >2% was found in 32% of NSCLC patients as early as 3 weeks after starting chemotherapy.^8^

Unfortunately, there are few examples in the literature of longitudinal body composition measurements in advanced NSCLC patients following initiation of systemic cancer treatment. Results of a meta‐analysis published in 2019 showed a significant decrease in muscle mass during chemotherapy in 2586 patients with multiple types of cancer.^9^ Three studies included in this report involved NSCLC patients, and one of these consisting of 35 patients found that some patients had increased muscle mass.^10^ Subsequent studies, evaluating both longitudinal muscle and adipose compartments in advanced NSCLC patients on chemotherapy, described decreases in mean cross‐sectional muscle area (SMA).^11^, ^12^ However, results for adipose tissue were not consistent. Decrease in mean cross‐sectional subcutaneous adipose, with no appreciable change in visceral adipose, was found in one study.^11^ In contrast, another group of investigators found increases in the cross‐sectional areas of both subcutaneous and visceral adipose tissues.^12^ Measurements were carried out at a single follow‐up point in both studies, and no data were reported for the percentage of patients who experienced gain in weight, muscle or adipose tissue.

Based on observations of weight gain during treatment and the relatively small amount of available longitudinal body composition data in NSCLC patients, we hypothesized that longitudinal body composition measurements might show increases in muscle mass and adipose tissue in some NSCLC patients during first line systemic therapy. The objectives of our study were to document the frequency of increases in skeletal muscle index (SMI), subcutaneous adipose tissue index (SATI), visceral adipose tissue index (VATI) and intramuscular adipose tissue (IMATI) and to evaluate the relationships between changes in these parameters and in body weight in two cohorts of advanced NSCLC patients during treatment with old versus new first line systemic cancer treatments.

## Methods

### Study populations

Two cohorts of ‘real world’ (not enrolled in a clinical trial) stage III/IV NSCLC patients receiving standard systemic cancer treatments as first line (i.e., treatment‐naïve) therapy were included in this study. No patients received dedicated cancer cachexia therapy or concurrent chest radiation. All patients provided written informed consent to our institutional review board approved protocol upon enrolment into the Rush University Cancer Center Biorepository and prior to selection for this study. Initially, we performed the analyses in stage IV NSCLC patients whose systemic cancer treatment consisted of newer platinum‐containing chemotherapy, anti‐PD1/PDL1 monoclonal antibodies alone or platinum‐containing chemotherapy combined with anti‐PD1/PDL1 monoclonal antibodies. These patients are classified as the current standard‐of‐care (CSOC) cohort, and their treatment was initiated between 16/12/2014 and 22/10/2020. Subsequently, we performed the same analyses in another cohort of stage III/IV NSCLC patients who received older platinum containing chemotherapy that was initiated between 16/6/2006 and 18/11/2014. They comprise the former standard‐of‐care cohort (FSOC). Our objective was to compare the consistency of longitudinal measurements of weight and body composition in the two cohorts. Each patient had measurement of body weight and height prior to first line systemic treatment (i.e., ‘baseline’), with body weight measured at the start of each subsequent cycle of systemic therapy. Chest computed tomography (CT) scans were accomplished prior to the first cycle and after the second (6 weeks) and fourth cycles (12 weeks) of systemic therapy. While a short course of corticosteroids (1–3 days) was routinely included in the anti‐emetic regimen for patients receiving platinum chemotherapy, prolonged corticosteroids or other potential cachexia treatments were not used. However, data regarding the use of concomitant medications were not included in the database for this study.

### Body composition measurements

Retrospective measurement of CT skeletal muscle cross‐sectional area at L1 was performed using semi‐automated software. Slice‐O‐Matic (version 3.0, TomoVision, Magog, Canada) + ABACS (Automated Body composition Analyzer using Computed tomography image Segmentation) module (https://www.voronoihealthanalytics.com/). Skeletal muscle cross‐sectional area was obtained at two consecutive axial images at the mid L1 vertebral body level. On each axial image, skeletal muscle was segmented automatically by the ABACS module and then reviewed by two independent observers (fellowship trained thoracic radiologists and CT trained body composition expert) using the Slice‐O‐Matic software. Skeletal muscle was segmented based on Hounsfield units (HU) with range of −29 to 150 HU; adipose tissues thresholds were segmented as follows: SAT and IMAT −190 to −30 HU; VAT −150 to −50 HU. The difference between the two SMAs at the same level was <1.5%. The final SMA was the average of the two readings. SMI was then calculated using the formula SMA (cm^2^) divided by height (m^2^). The L1 vertebral body level was selected for evaluation of body composition analysis, given our routine chest CT scans for patient restaging did not include the L3 vertebral body level. Indices for the other body composition parameters (SATI, VATI and IMATI) were calculated in an identical fashion.

### Study objectives and data analysis plan

The first objective of this study was to determine, and to compare, the frequency of weight gain and body composition parameter (i.e., SMI, SATI, VATI and IMATI) increases in the CSOC and FSOC patient cohorts. The second objective was to evaluate, and to compare, correlations between body weight and body composition changes in each patient cohort.

All statistical analyses were performed using the R software (www.r‐project.org). Variables were evaluated as both continuous and categorical scales and summarized as median (or mean) values with range, or number and percentage of cohort, provided. Group comparisons for continuous and binary variables were performed using the Mann–Whitney rank–sum (two‐sided) test, Chi‐square test and Fisher's exact test, respectively. Association between weight change and body composition changes were measured by Pearson correlation. The correlations of weight and body composition between CSOC and FSOC cohorts were compared by two‐sample tests for correlations. Overall survival (OS) was measured from date of initiation of systemic cancer treatment until death from any cause. Progression‐free survival (PFS) was measured from date of initiation of cancer treatment until cancer progression or death from any cause. Disease progression was defined according to the following considerations. We reviewed images and reports from diagnostic studies (CT scans, magnetic resonance imaging scans and positron emission scans), as well as physician notes describing the patient's disease status (responding, stable disease, vs. progression), In addition, discontinuation of first line systemic therapy and the need for radiotherapy to palliate symptoms from disease progression were considered in classifying a patient as having progressive disease. Strict RECIST criteria were not used. OS and PFS were estimated using the Kaplan–Meier method and compared between groups using the log‐rank test.

## Results

This study included retrospective measures of weight, BMI, and body composition in CSOC (106 patients) and FSOC (88 patients) cohorts of advanced NSCLC patients who were enrolled in our institutional biorepository and received first line systemic cancer regimens. Baseline patient characteristics are shown in *Table* *1*. Chi‐square was used to compare differences in baseline parameters between the two cohorts. There were no significant differences in sex, race, weight, BMI, and BMI categories between the cohorts. However, the CSOC cohort was older (median age: 68 vs. 64 years, *P* < 0.001) and had a higher percentage of patients with adenocarcinoma histology (80.6% vs. 61.2%, *P* = 0.006). The FSOC cohort included stage III patients (11.4%) while the CSOC cohort included only stage IV patients. Stage III patients in the FSOC cohort did not receive chest radiation.

**Table 1:** Baseline patient characteristics

Parameter		Current standard‐of‐care cohort (*n* = 106)	Former standard‐of‐care cohort (*n* = 88)	*P* values
Age, years	median (IQR)	69 (12)	64 (15)	<0.001
Weight, kg	median (IQR)	74.21 (26.49)	71.91 (19.18)	0.271
BMI, kg/m^2^	median (IQR)	25.63 (6.93)	25.97 (6.29)	0.846
BMI groups				0.745
<20 kg/m^2^	number (%)	14 (13.2%)	10 (11.4%)	
≥20 < 25 kg/m^2^	number (%)	34 (32.1%)	26 (29.5%)	
> 25 < 30 kg/m^2^	number (%)	39 (36.8%)	39 (44.9%)	
≥ 30 kg/m^2^	number (%)	19 (17.9%)	13 (14.8%)	
SMI, cm^2^/m^2^	median (IQR)	37 (12)	36 (11)	0.924
SATI, cm^2^/m^2^	median (IQR)	35.1 (39.5)	33.9 (32.6)	0.623
VATI, cm^2^/m^2^	median (IQR)	37.1 (36.6)	26.7 (35.7)	0.016
IMATI, cm^2^/m^2^	median (IQR)	5.4 (3.5)	5.17 (4.7)	0.599
Sex				0.153
Males	number (%)	58 (53.7%)	38 (43.2%)	
Females	number (%)	50 (46.3%)	50 (56.8%)	
Race				1.0
White	number (%)	74 (68.5%)	62 (70.4%)	
Black	number (%)	27 (25.0%)	22 (25.0%)	
Asian/Pacific Islander	number (%)	2 (1.8%)	4 (4.6%)	
Unknown	number (%)	5 (4.7%)	0 (0%)	
Cancer stage				
IIIA/B	number (%)	0 (0%)	10 (11.4%)	
IV	number (%)	108 (100%)	78 (88.6%)	
Histology				
Adenocarcinoma	number (%)	87 (80.6%)	55(62.5%)	0.006
Squamous cell cancer	number (%)	14 (13.0%)	20(22.7%)	
Non‐small cell cancer	number (%)	7 (6.4%)	13(14.8%)	
ECOG PS				
0/1	number (%)	90 (83.3%)	Not available	
2	number (%)	18 (16.7%)	Not available	
Smoking history				
Current/former	number (%)	99 (91.7%)	Not available	
Never	number (%)	9 (8.3%)	Not available	
Treatment regimens				
Platinum combo Chemo	number (%)	34 (31.5%)	88(100%)	
Platinum combo Chemo + anti‐PD1/L1 mAbs	number (%)	46 (42.6%)	None	
Anti‐PD‐1/‐L1 mAbs	number (%)	27 (35.9%)	None	

*Note*: Group comparisons for continuous and categorical variables were performed using the Mann–Whitney rank–sum (two‐sided) test and Chi‐square test, respectively.

Abbreviations: BMI, body mass index; ECOG, Eastern Cooperative Oncology Group; IMATI, intramuscular adipose tissue; SATI, subcutaneous adipose tissue index; SMI, skeletal muscle index; VATI, visceral adipose tissue index.

Significant differences between cohorts were not found in baseline SMI, SATI and IMATI values, measured at L1. The CSOC cohort had a higher median baseline level of VATI (36.9 vs. 26.7 cm^2^/m^2^, *P* = 0.016). In the CSOC cohort, 91.7% had a current or former smoking history and 16.7% were Eastern Cooperative Oncology Group (ECOG) performance status 2. Data for smoking history and performance status were not available for the FSOC cohort.

**Figure 1:**
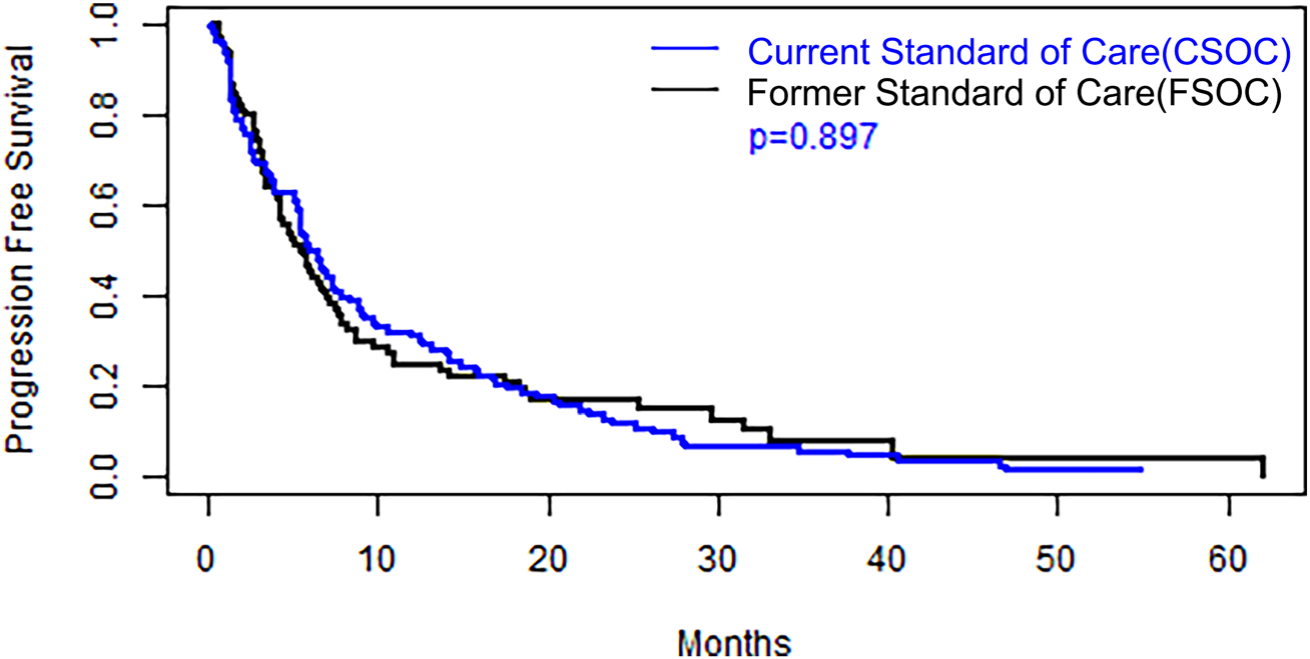
Kaplan–Meier estimates of progression free survival for the current standard‐of‐care and former standard‐of‐care cohorts and *P* value for group comparison based on a log‐rank test.

**Figure 2:**
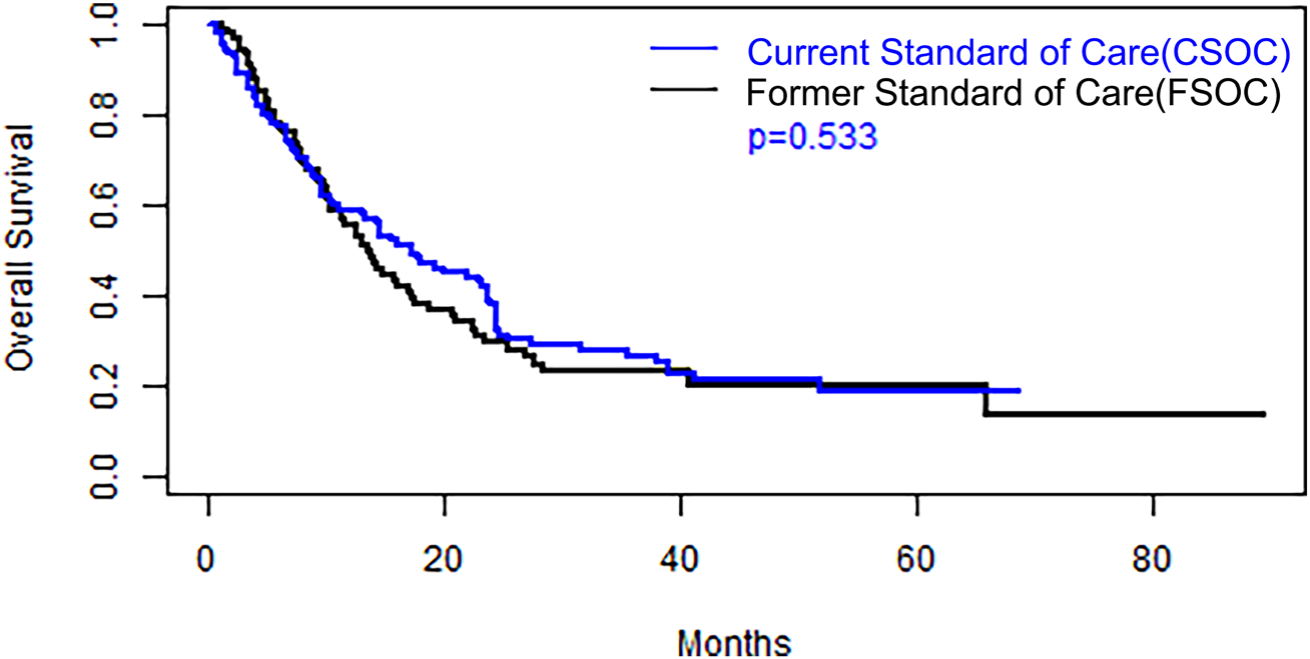
Kaplan–Meier estimate of overall survival for the current standard‐of‐care and former standard‐of‐care cohorts and *P* value for group comparison based on a log‐rank test.

As previously stated, the cohorts differed in the types of treatment regimens received. The FSOC cohort patients were treated with platinum‐containing chemotherapy only. The CSOC group patients were treated with platinum containing regimens (31.5%), anti‐PD1/PDL1 monoclonal antibodies (35.9%) and platinum containing chemotherapy combined with anti‐PD1/PDL1 monoclonal antibodies (42.6%). Kaplan–Meier plots for PFS and OS for both cohorts are depicted in *Figures* *1* and *2*, respectively. The median PFS duration was 6.05 months for the CSOC group, versus 5.49 months for the FSOC group (hazard ratio = 1.02, *P* = 0.90). The median OS durations were 17.3 months for the CSOC cohort and 13.5 months for the FSOC cohort (hazard ratio = 0.9, log‐rank *P* = 0.53).

Patients in the CSOC cohort had a greater frequency of missing weight, BMI and body composition measurements at the 6 and 12 week measurements (*Table* *2*). Similarly, there is a higher percentage of missing body composition measurements in the CSOC cohort in the analyses evaluating correlations between weight and body composition changes (*Table* *3*). Missing measurements did not inflate the percentage of patients who experienced weight or body composition increases of ≥2.5% or >5% because the total number of patients (106 and 88 patients) was used as the denominator in these calculations.

**Table 2:** Longitudinal body weight/BMI and body composition changes relative to baseline

Parameter		*n* (% missing)	Current standard‐of‐care cohort	*n* (% missing)	Former standard‐of‐care cohort	*P* values
6 Week changes						
Weight change (kg)	mean (*SD*)	93 (12.3%)	−0.87 (3.22)	88 (0.0%)	−1.45 (3.22)	0.222
BMI change (kg/m^2^)	mean (*SD*)	93 (12.3%)	−0.29 (1.06)	88 (0.0%)	−0.52 (1.13)	0.192
SMI change (cm^2^/m^2^)	mean (*SD*)	66 (37.7%)	−1.22 (−3.78)	83 (5.7%)	−1.73 (3.33)	0.498
SATI change (cm^2^/m^2^)	mean (*SD*)	66 (37.7%)	−3.19 (6.88)	69 (21.6%)	−2.95 (13.09)	0.072
VATI change (cm^2^/m^2^)	mean (*SD*)	66 (37.7%)	−1.6 (9.36)	86 (2.3%)	0.91 (8.06)	0.018
IMATI change (cm^2^/m^2^)	mean (*SD*)	66 (37.7%)	0.35 (1.13)	86 (2.3%)	0.11 (1.17)	0.256
Weight gain ≥2.5%	number (%)	106(0%)	16 (14.8%)	88 (0.0%)	13 (14.8%)	0.839
BMI gain ≥2.5%	number (%)	106(0%)	16 (14.8%)	88 (0.0%)	13 (14.8%)	0.844
SMI gain ≥2.5%	number (%)	106(0%)	20 (18.5%)	88 (0.0%)	15 (18.2%)	0.542
SATI gain ≥2.5%	number (%)	106(0%)	32 (30.4%)	88 (0.0%)	28 (31.8%)	0.96
VATI gain ≥2.5%	number (%)	106(0%)	44 (40.1%)	88 (0.0%)	49 (55.7%)	0.37
IMATI gain ≥2.5%	number (%)	106(0%)	50 (46.3%)	88 (0.0%)	46 (52.3%)	1.0
Weight gain ≥5%	number (%)	106(0%)	4 (3.7%)	88 (0.0%)	4 (4.5%)	1.0
BMI gain ≥5%	number (%)	106(0%)	4 (3.7%)	88 (0.0%)	4 (4.5%)	0.242
SMI gain ≥5%	number (%)	106(0%)	17 (15.7%)	88 (0.0%)	11 (12.5%)	0.196
SATI gain ≥5%	number (%)	106(0%)	28 (25.9%)	88 (0.0%)	24 (27.3%)	0.273
VATI gain ≥5%	number (%)	106(0%)	41 (38.1%)	88 (0.0%)	47 (53.4%)	0.076
IMATI gain ≥5%	number (%)	106(0%)	47 (43.5%)	88 (0.0%)	38 (43.2%)	0.518
12 Week changes						
Weight change (kg)	mean (*SD*)	88 (17.0%)	−0.05 (5.87)	83 (5.7%)	−1.90 (4.91)	0.055
BMI change (kg/m^2^)	mean (*SD*)	88 (17.0%)	−0.19 (1.5)	83 (5.7%)	−0.67 (1.73)	0.52
SMI change (cm^2^/m^2^)	mean (*SD*)	60 (43.4%)	−1.2 (3.89)	71 (19.3%)	−1.73 (3.54)	0.412
SATI change (cm^2^/m^2^)	mean (*SD*)	60 (43.4%)	−0.92 (9.2)	57 (35.2%)	−2.45 (11.82)	0.872
VATI change (cm^2^/m^2^)	mean (*SD*)	60 (43.4%)	1.50 (11.88)	72 (18.2%)	1.84 (10.85)	0.933
IMATI change (cm^2^/m^2^)	mean (*SD*)	60 (43.4%)	0.23 (1.22)	72 (18.2%)	0.46 (1.71)	0.474
Weight gain ≥2.5%	number (%)	106(0%)	28 (25.9%)	88 (0.0%)	18 (20.5%)	0.168
BMI gain ≥2.5%	number (%)	106(0%)	28 (25.9%)	88 (0.0%)	18 (20.5%)	0.168
SMI gain ≥2.5%	number (%)	106(0%)	25 (23.1%)	88 (0.0%)	17 (19.3%)	1.0
SATI gain ≥2.5%	number (%)	106(0%)	28 (25.9%)	88 (0.0%)	24 (27.3%)	1.0
VATI gain ≥2.5%	number (%)	106(0%)	41 (38.1%)	88 (0.0%)	36 (40.9%)	0.485
IMATI gain ≥2.5%	number (%)	106(0%)	51 (47.2%)	88 (0.0%)	45 (51.1%)	0.595
Weight gain ≥5%	number (%)	106(0%)	13 (12.0%)	88 (0.0%)	7 (8.0%)	0.242
BMI ≥ 5%	number (%)	106(0%)	13 (12.0%)	88 (0.0%)	7 (8.0%)	0.231
SMI gain ≥5%	number (%)	106(0%)	14 (13.0%)	88 (0.0%)	9 (10.2%)	0.797
SATI gain ≥5%	number (%)	106(0%)	25 (23.1%)	88 (0.0%)	22 (25.0%)	0.711
VATI gain ≥5%	number (%)	106(0%)	39 (36.0%)	88 (0.0%)	33 (37.5%)	0.861
IMATI gain ≥5%	number (%)	106(0%)	49 (45.4%)	88 (0.0%)	42 (47.7%)	0.483

*Note*: Group comparisons for continuous and binary variables were performed using the Mann–Whitney rank–sum (two‐sided) test and Fisher's exact test, respectively. Calculations of mean changes in the parameters are based on the number of patients who had measurements at the 6 and 12 week time points. Calculation of the percentages of patients with weight, BMI, and body composition increases >2.5% and >5% are based on the total number of patients at baseline and are reflected in the column that shows 0% missing values for these parameters.

Abbreviations: BMI, body mass index; IMATI, intramuscular adipose tissue; SATI, subcutaneous adipose tissue index; SMI, skeletal muscle index; VATI, visceral adipose tissue index.

**Table 3:** Correlations between body weight change and changes in muscle mass and adipose tissue over 12 weeks

	Current standard‐of‐care cohort (*n* = 106 patients)			Former standard‐of‐care cohort (*n* = 88 patients)			
Time point muscle/fat change	*n* (% missing)	Pearson correlation	*P* value	*n* (% missing)	Pearson correlation	*P* value	Cohort comparison *P* value
6 Weeks							
SMI change	60 (43.4%)	0.44	<0.001	83 (5.7%)	0.48	<0.001	0.79
SATI change	60 (43.4%)	0.31	0.02	69 (21.6%)	0.39	0.001	0.63
VATI change	60 (43.4%)	0.04	0.79	86 (2.3%)	0.36	<0.001	0.05
IMATI change	60 (43.4%)	0.32	0.01	86 (2.3%)	0.14	0.21	0.27
12 Weeks							
SMI change	57 (46.2%)	0.58	<0.001	70 (34.0%)	0.49	<0.001	0.49
SATI change	57 (46.2%)	0.32	0.02	56 (47.2%)	0.53	<0.001	0.20
VATI change	57 (46.2%)	0.37	0.005	71 (33.0%)	0.37	0.001	0.98
IMATI change	57 (46.2%)	0.26	0.049	71 (33.0%)	0.47	<0.001	0.18

*Note*: Fisher's test for two correlations was used to compare the two cohorts at 6 and 12 weeks.

Abbreviations: BMI, body mass index; IMATI, intramuscular adipose tissue; SATI, subcutaneous adipose tissue index; SMI, skeletal muscle index; VATI, visceral adipose tissue index.

Mean changes in weight/BMI, the frequency of increases in weight/BMI and potential differences in these parameters between the cohorts are shown in *Table* *2*. At the 6 week follow‐up time point, both cohorts had decreases in mean weight (CSOC cohort = −0.87 kg; FSOC = −1.45 kg, *P* = 0.222). Mean BMI change was −0.29 kg/m^2^ in the CSOC group and −0.52 kg/m^2^ in the FSOC group (*P* = 0.192). At the same time point, 14.8% of patients in both groups had ≥2.5% weight gain and ≥2.5% BMI increase. The frequencies of weight/BMI increases ≥5% at 6 weeks were 3.7% and 4.5% for the CSOC and FSOC cohorts, respectively (*P* = 1.0).

The mean weight change at 12 weeks in the CSOC group was −0.51 kg versus −1.90 kg in the FSOC group (*P* = 0.055). Similar results were observed for BMI with the mean change being −0.18 kg/m^2^ in the CSOC cohort and −0.67 kg/m^2^ in the FSOC cohort (*P* = 0.52). Weight/BMI gain ≥2.5% at 12 weeks occurred in 25.9% in the CSOC cohort and in 20.5% of the patients in the FSOC cohort (*P* = 0.168). A smaller number of patients experienced ≥5% weight/BMI gain at 12 weeks; 12% in the CSOC cohort and 8% in the FSOC cohort.

Body composition changes that occurred 6 and 12 weeks after initiation of systemic cancer treatment are also shown in *Table* *2*. Mean SMI change at 6 weeks was −1.2 cm^2^/m^2^ in the CSOC cohort versus −1.73 m^2^/m^2^ in the FSOC cohort (*P* = 0.498). Mean SMI changes in the cohorts at 12 weeks were identical to the results at 6 weeks (*P* = 0.412). The percentages of patients who experienced ≥2.5% SMI increase at the 6 week follow‐up were 18.5% in the CSOC group and 18.2% in the FSOC group (*P* = 0.542). At 12 weeks, >2.5% SMI increase occurred in 23.1% of patients in the CSOC cohort versus 19.3% in the FSOC cohort (*P* = 1.0). SMI increases >5% were found at 6 weeks: 15.7% (CSOC cohort) versus 12.5% (FSOC cohort; *P* = 0.196), and the frequency of SMI increases >5% were similar at 12 weeks: 13.0% (CSOC cohort) versus 10.2% (FSOC cohort; *P* = 0.797).

Findings for longitudinal SATI measurements are also depicted in *Table* *2*. Decreases in mean SATI occurred in both groups with mean SATI changes of −3.19 and −2.95 cm^2^/m^2^ at 6 weeks (*P* = 0.072) for the CSOC and FSOC cohorts, respectively. Similar changes were seen at the 12 week time point with mean SATI changes of −0.92 and −2.45 cm^2^/m^2^ (*P* = 0.872). Increases in SATI of ≥2.5% occurred at 6 weeks in 30.4% (CSOC) and 31.8% (FSOC) of patients (*P* = 0.96) and at 12 weeks in 25.9% (CSOC) and 27.3% (FSOC) of patients (*P* = 1.0). SATI increases ≥5% were observed in 25.9% (FSOC) and 27.3% (CSOC) of patients at 6 weeks (*P* = 0.273), and in 23.1% (CSOC) and 25.0% of patients (FSOC) at 12 weeks (*P* = 0.711).

At 6 weeks, the mean VATI change in the CSOC cohort was −1.6 cm^2^/m^2^, and in the FSOC cohort it was +0.91 cm^2^/m^2^ (*P* = 0.018) (*Table* *2*). Both cohorts experienced mean increase in VATI at 12 weeks: +1.50 cm^2^/m^2^ (CSOC) and +1.84 cm^2^/m^2^(FSOC) (*P* = 0.933). VATI increases of ≥2.5% were found in 40.1% (CSOC) and 55.7% (FSOC) of patients (*P* = 0.37) at 6 weeks and in 38.1% (CSOC) and 40.9% (FSOC) of patients at 12 weeks (*P* = 0.485). Six‐week VATI increases ≥5% occurred in 38.1% of patients in the CSOC cohort and in 53.4% of the FSOC cohort (*P* = 0.076). Both groups had ≥5% VATI increases at 12 weeks: 36.0% and 37.5% (*P* = 0. 861).

Increases in mean IMATI changes were observed in both groups at 6 and 12 weeks with the gains ranging from +0.11 to +0.46 cm^2^/m^2^ (*P* = 0.256 at 6 weeks and *P* = 0.474 at 12 weeks). IMATI increases ≥2.5% at 6 and 12 weeks occurred in 46.3–52.3% of the patients in both groups, and there were no significant differences between the cohorts (*Table* *2*). The percentages of patients with IMATI gains of ≥5% in both groups varied from 43.2% to 47.7%. Differences between the cohorts were not significant.

Correlations between weight change and body composition are shown in *Table* *3*. In both cohorts, there were direct correlations between SMI change and weight change at 6 and 12 weeks. Pearson correlation coefficients varied between 0.44 and 0.58 overall, and *P* values for the SMI Pearson coefficients were <0.001. Evaluation of the relationship between changes in weight and in SATI also demonstrated a positive relationship in both cohorts at the 6 and 12 week time points with Pearson coefficients varying between 0.31 and 0.53, with *P* values varying <0.02. Comparison of the Pearson coefficients for weight and SMI and for weight and SATI for the CSOC versus the FSOC cohort revealed no significant differences at the 6 or 12 week time points.

Results for associations between VATI change and weight change are also shown in *Table* *3*. There was a direct correlation between weight and VATI changes in both cohorts at the 6 and 12 week time points. Pearson coefficients varied from 0.04 to 0.37, and *P* values were <0.005 except for the 6 week measurement in the CSOC cohort. Similarly, comparisons of the Pearson coefficients for each cohort were not significantly different except for the 6 week time point. The Pearson coefficients for IMATI/weight changes in the FSOC patients were 0.14 (*P* = 0.21) and 0.47 (*P* < 0.001) at 6 and 12 weeks, respectively. In the CSOC group, the Pearson coefficient at 6 weeks was 0.32 (*P* = 0.01), and the Pearson coefficient at 12 weeks was 0.26 (*P* = 0.049). Comparison of the Pearson coefficients for each cohort showed no significant differences.

## Discussion

Weight gain that was observed in a subset of our real‐world NSCLC patients has been described previously in clinical trial NSCLC patients who received platinum‐based chemotherapy.^7^, ^8^ However, our results, utilizing CT scans to determine body composition at the L1 level, appear to be the first description of increases in both muscle mass and adipose tissue in a subset of NSCLC patients while receiving cancer treatment. Because cytotoxic treatments have been associated with loss of muscle mass,^13^ increases in SMI in two NSCLC patient cohorts were particularly unexpected. Measuring body composition at the L3 level on CT scans has been shown to be superior to bioelectrical impedance analysis and is as accurate as dual X‐ray absorptiometry.^14^ Body composition measurements at other spinal levels including L1 have also been suggested as viable landmarks in cancer patients^14^ and in healthy individuals.^15^ Because our patients had chest CT scans that did not include L3 levels and our objective was to evaluate body composition changes over time, we considered measurement at the L1 level to be a valid methodology. In addition, body composition measurements were quantified with the more recently developed Automated Body composition Analyzer using Computed tomography image Segmentation (ABACS) software previously validated on approximately 6000 early‐stage colon and early‐stage breast cancer patients.^15^

### Weight gain changes

Weight gain has been described in a subset of advanced NSCLC patients during treatment with first line platinum containing chemotherapy.^6^, ^7^, ^8^ These studies involved post‐hoc analyses that included 882 to 2301 NSCLC patients who had participated in clinical trials. The current study included a cohort of patients who received the FSOC that consisted only of older platinum chemotherapy regimens and a patient cohort that received the CSOC that included three types of treatment: anti‐PD1/PDL1 monoclonal antibodies only, anti‐PD1/PDL1 monoclonal antibodies plus platinum chemotherapy, or only platinum chemotherapy. The use of immunotherapy alone was based on the phase III trial that showed pembrolizumab was associated with superior OS compared with platinum chemotherapy in NSCLC patients whose tumours had high PDL1 expression.^16^ A subset of CSOC patients were treated with immunotherapy and concurrent chemotherapy based on results of a phase III trial, which showed superior OS in patients treated with immunotherapy plus chemotherapy versus chemotherapy only.^17^ It is possible that the results from our study might not be representative of results from a larger patient population though we did not detect major differences in our cohort findings despite the inclusion of immune checkpoint inhibition in our CSOC cohort. Also, we considered that our results might be atypical for real world advanced stage NSCLC patients. Therefore, we compared our observations regarding the frequency of either weight gain or mean changes in body composition with results described in previous reports to look for major inconsistencies.

The findings from our study and observations described in larger NSCLC studies are relatively similar.^6^, ^7^, ^8^ In one of the earlier reports,^8^ 11.7% of patients experienced weight gain >5% during the first 12 weeks compared with 12% in our CSOC cohort and 8% in our FSOC cohort during the same time period. Another group of investigators found that a higher number of patients (18.2%) had weight gain >5%.^7^ They included weight measurements beyond 12 weeks, and it is possible that their observation of a higher frequency of >5% weight gain might be related to the inclusion of weight determinations at later time points.^7^

In a large study that evaluated weight loss and BMI in patients with multiple types of cancer, the investigators recommended that stable weight be defined as ±2.4% based on mean diurnal weight variation of 0.2%.^18^ Consistent with this recommendation, Roeland et al.^8^ evaluated the frequency of weight gain >2.5%, as well as weight gain >5%.^8^ This group of investigators found that 24.5% of the patients had >2.5% weight gain at 3 months. Similar results were observed at 12 weeks in our study with weight gain ≥2.5% observed in 25.9% of the CSOC cohort patients and in 20.5% of our FSOC cohort patients. Chi‐square analysis showed that results in our cohorts were not significantly different (*P* = 0.168) (*Table* *2*).

It appears that weight gain occurs early in some advanced NSCLC patients. Although weight gain was not defined as a specific percentage, investigators found that 208 of 656 (32%) NSCLC patients had gained some weight at the time that they received their second cycle of chemotherapy, presumably 3 to 4 weeks after treatment initiation.^6^ Relatively early weight gain was also observed in our study with 14.8% of patients having ≥2.5% weight gain at 6 weeks in both cohorts.

### Skeletal muscle changes

There are at least seven studies that reported results for muscle changes between baseline and follow measurements in stage IV NSCLC patients during treatment with first line chemotherapy.^10^, ^11^, ^12^, ^19^, ^20^, ^21^, ^22^ SMI calculations were based on cross‐SMA at the level of L3 in all studies. Each study was relatively small. The collective number of patients included in these reports was 591, and the number varied from 30 patients^22^ to 200 patients.^11^ All these investigators reported overall losses in muscle mass during chemotherapy, and four groups^11^, ^12^, ^21^, ^22^ reported results for mean SMI changes that ranged from −1.2 to −1.8 cm^2^/m^2^. Similarly, a large study in surgically treated stage I‐III NSCLC patients found significant muscle mass decrease at the time of lung cancer recurrence. The mean change in muscle mass was not described in this study.^23^ Decreases in mean SMI at 6 and 12 weeks in our patients ranged from −1.20 to −1.73 cm^2^/m^2^ and are similar to SMI changes in the stage IV NSCLC studies cited above.^10^, ^11^, ^12^, ^17^, ^18^, ^19^, ^20^

Results of follow‐up muscle mass measurements have also been described in advanced NSCLC patients who were receiving targeted therapies (EGFR or ALK tyrosine kinase inhibitors)^20^ or who were receiving immunotherapy (i.e., anti‐PD1/PDL1 monoclonal antibodies).^24^, ^25^ The targeted therapy study was small, and the study did not report mean or categorical SMI decreases. However, the investigators found less muscle mass decrease in patients treated with targeted therapies versus muscle loss in chemotherapy treated patients.^20^ Loss of muscle mass was also observed in NSCLC patients treated with first‐ or second‐line immunotherapy (pembrolizumab or nivolumab). Although the mean change in SMI was not reported in this study,^25^ 15 of 61 patients experienced >5% SMI decrease 8 weeks after starting immunotherapy. Another group of investigators^24^ also found >1.3% muscle mass decrease in 42 of 80 (52.5%) patients treated with nivolumab.

None of the above reports^10^, ^11^, ^12^, ^21^, ^22^, ^23^ described the frequency of SMI gain defined as a categorical variable. However, either stabilization or any increase in SMI was observed in 16 of 35 NSCLC patients in one of the studies.^10^ Similarly, another report described an undefined level of SMI increase in 17 of 58 (30%) advanced NSCLC patients during treatment with chemotherapy.^22^ Our observations that 18.2% to 23.1% of the patients in both cohorts had ≥2.5% SMI gains and that 10.2% to 15.7% of the patients in both cohorts had ≥5% SMI increases appear to be the first description of a specifically defined increase in muscle mass in advanced NSCLC patients.

Having observed some increase in SMI in a small number of NSCLC patients during chemotherapy, Stene et al.^10^ have suggested that cancer treatment could inhibit factors that are driving muscle breakdown and cancer cachexia. SMI and weight increase during systemic cancer treatment observed in subsets of our patients is consistent with their hypothesis. Patel et al.^7^ found that the objective response rate on chemotherapy was 50% in patients whose weight gain was ≥5% versus a 25% response rate in patients whose weight gain was <5%, which also suggests that treatment‐related tumour regression might reduce mechanisms that promote cachexia. However, these findings fail to address the question of whether systemic cancer treatment can reverse ongoing muscle breakdown and weight loss. Exploration of this hypothesis would require determination of SMI and weight trajectories before and after initiation of systemic cancer treatment.

### Adipose tissue changes

Two of the studies described above included data for follow‐up adipose tissue measurements, as well as muscle mass measurements.^11^, ^12^ With a mean follow‐up measurement time of 4.4 months, Nattenmuller et al.^11^ observed increases in visceral and subcutaneous adipose tissue cross‐sectional area (+9.1 and +5.9 cm^2^) and an increase of IMATI of +2.0 cm^2^/m^2^ in NSCLC patients being treated with chemotherapy. Conflicting results regarding subcutaneous adipose tissue change were reported by another group of investigators.^12^ They found a mean SATI change of −1.9 cm^2^/m^2^ in 111 NSCLC patients 6 weeks after starting chemotherapy. Mean SATI changes in our patients at 6 and 12 weeks varied from −0.92 to −3.19 cm^2^/m^2^ in both cohorts and are similar to results reported by Degens et al.^12^ Like Nattenmuller et al.,^11^ we also observed increases in mean visceral adipose tissue at the 12 week follow‐up point (*Table* *2*). These investigators^11^ also found increases in intramuscular adipose tissue with mean cross‐sectional area of +2 cm^2^, and we observed mean IMATI increases of +0.11 to +0.46 cm^2^/m^2^ (*Table* *2*).

In addition to describing mean measurements in adipose tissue at two time points, we evaluated the percentages of patients who experienced gains in adipose tissue at the same time points. We found that adipose tissue (SATI, VATI, IMATI) increases occurred more frequently than muscle mass (SMI). At the 12 week time point, between 25.9% and 51.1% of patients had >2.5% adipose tissue increases compared with 19.3% to 23.1% of patients who had SMI increases ≥2.5% (*Table* *2*). Adipose tissue increases of ≥5% were also more frequent than SMI increases of ≥5% at the 12 week measurement (*Table* *2*).

There is a possibility that the amount of adipose tissue might play a role in biologic processes involved in NSCLC patients' outcomes and efficacy of immunotherapy. Although obesity is associated with increased all‐cause mortality,^26^ high BMI was associated with superior OS in advanced^27^ and in early stage^28^ NSCLC patients and with longer survival in cancer patients treated with immune checkpoint inhibitors.^13^, ^29^ These observations, and our finding for increases in adipose tissue compartments in some of our patients, suggest that additional study of longitudinal adipose tissue measurements in patients with NSCLC and with other types of cancer patients is warranted.^11^, ^24^, ^30^

One previously published study examined changes in subcutaneous and visceral adipose tissue and weight loss. These investigators found that weight loss >2% was associated with visceral adipose tissue loss (*P* = 0.047) and with superficial adipose tissue loss (*P* = 0.042).^24^ Weak direct relationships between weight and SATI changes and between weight and VATI changes were also observed in our patients with the majority of Pearson coefficients being less than 0.40 (*Table* *3*). Similar relationships between weight and IMATI changes were also observed. The correlations between weight, VATI and IMATI were less consistent than correlations between weight and SMI and weight and SATI (*Table* *3*). Our findings provide preliminary evidence that weight gain, in a subset of NSCLC patients who are receiving systemic cancer treatment, is associated with increases in muscle and in subcutaneous, visceral and intramuscular adipose tissue.

### Study weaknesses

Weaknesses in our study include the small number of patients in each cohort, the retrospective collection of clinical data and body composition changes, the relatively high number of missing body composition measurements in the CSOC cohort, the differences between the cohorts in systemic cancer treatment regimens. We are hopeful that increasing awareness of cachexia, increasing use of electronic medical records and the availability of automated body composition measurements will reduce the amount of missing data in real world NSCLC patients and patients with other types of cancer. We believe that the major weakness of our study is the absence of data regarding pre‐treatment weight loss. Therefore the diagnosis of baseline cachexia as defined by International Consensus^31^ was not feasible. Increase in weight was observed in a subset of patients, but we do not know whether weight gain occurred in patients who were losing weight prior to starting cancer treatment. Similarly, data for body composition prior to starting cancer were not available. Despite these limitations, the frequencies of increases in weight and body composition in both cohorts are consistent and suggest that further study of these parameters in a larger number of real‐world patients and in cachexia clinical trials should be considered.

### Future directions and implications

The complexity of conducting cancer cachexia clinical trials has been comprehensively reviewed by Le‐Rademacher et al.^32^ The findings in our patients could add to the complicated considerations involved in designing cachexia clinical trials. Results from Phase III placebo‐controlled trials in advanced NSCLC patients showed that anamorelin^33^ and enobosarm^34^ were associated with significant increases in lean body mass despite significant differences in eligibility criteria. The anamorelin trial included patients who had multiple types of cancer treatment including first line chemotherapy, greater‐than second line chemotherapy, radiation therapy only or best supportive care only. In contrast, the enobosarm trial included only treatment‐naïve NSCLC patients who received the same platinum regimen. When these studies^33^, ^34^ were conducted, the available NSCLC treatments regimens were relatively ineffective. More effective NSCLC treatment regimens are available now. Significantly higher tumour response rates and superior OS have been observed with immunotherapy regimens^17^, ^35^ and therapies that target specific tumour mutations.^36^, ^37^ Increased frequency of gains in weight, muscle mass and adipose tissue were not observed in our CSOC cohort of patients (*Table* *2*) despite the fact that 70% of them were treated with immunotherapy regimens. However, it is conceivable that newer cancer treatments might be associated with more frequent weight and body composition increases. On the premise that more effective cancer treatment is associated with significant increases in weight and body composition, the type of cancer regimen should be a stratification factor in randomized NSCLC cachexia trials. Also, the possibility that more effective cancer treatment regimens are associated with higher frequency of weight and body composition increases in placebo treated patients in randomized cachexia trials has implications for calculating sample size in these studies.

Comparison of patients with increases in weight and body composition versus patients with progressive cachexia could also have implications for translational research. Baracos^38^ has discussed the complex relationships between altered energy balance, altered central nervous system homeostasis, muscle loss, and adipose tissue depletion that occur in cachexia. Circulating catabolic factors secreted by tumours and circulating mediators of inflammation that arise from the interaction of tumour and immune cells appear to be driving the tissue alterations involved in cachexia. However, the mechanisms driving cachexia and the relationship between weight and body composition are not fully understood. In addition, it is likely that heterogeneous factors are driving cachexia and that individualized treatment strategies will be required. Currently, there is software that enables rapid measurement of body composition^15^ on routine CT scans, and there is multiplex serum protein platforms^39^, ^40^ that analyse serum proteins from an additional tube of blood obtained at the time of routine follow‐up blood tests. Comparison of results obtained with these technologies in patients with progressive cachexia versus those with reversal of cachexia during cancer treatment could provide insights regarding mechanisms driving cachexia and could lead to identification of novel therapeutic targets.

## Conclusion

In summary, the frequency of weight gain observed in two small cohorts of advanced NSCLC patients receiving first line systemic therapy was similar to the frequency found in larger advanced NSCLC studies. Subsets of patients in both of our cohorts had increases in muscle mass and in adipose tissue, and there were weak to moderate direct correlations between changes in weight and in body composition with stronger correlations observed between weight and muscle mass. If these findings are confirmed in larger studies, they could have implications for clinical trial design and for translational research in cachectic and pre‐cachectic advanced NSCLC patients, and possibly in patients with other malignancies.

## Ethics statement

This study was approved by the Institutional Review Board at Rush University Medical Center, and all subjects provided written informed consent prior to inclusion. Therefore, this study was performed in accordance with the ethical standards laid down in the 1964 Declaration of Helsinki and its later amendments.

## Conflicts of interest statement

Sanjib Basu has received honoraria from Eli Lilly for serving as a consultant. Philip Bonomi has received honoraria from Helsinn, Pfizer and Roche Genentech for participation in advisory boards and in an independent data monitoring committee. Jeffrey Borgia has received research funding from Pfizer, Abbott Laboratories, Serum Inc., Biodesix, Epigenomics and Tempus Laboratories and serves on the scientific advisory boards for Luminex Corporation and Rational Vaccines. Mary Jo Fidler has received honoraria for participation in the following advisory boards: AbbVie, Astra Zeneca, Beigene, Biodesix, Bluprint, Bristol Myers Squibb, Daiichi, EMD Serono, G1 Therapeutics, Genentech, Gilead, Jannsen, Jazz Pharma, Oncohost, Regeneron, Silverback, Takeda and Tempus. Sandra Gomez has no conflicts. Palmi Shah has no conflicts. Marta Batus has no conflicts. Levi B. Martinka has no conflicts. Ahmed Abdelkader has no conflicts. Iphigenia Tzameli is an employee of Pfizer Inc. Sonia Cobain is an employee and shareholder of Pfizer Inc. Susie Collins is an employee and shareholder of Pfizer Inc. Ned Keliher is an employee of Pfizer Inc. Danna M Breen is an employee and shareholder of Pfizer Inc. Julia Brosnan is an employee and shareholder of Pfizer Inc. Roberto A. Calle was an employee of Pfizer Inc. at the time of the study and is a Pfizer and Regeneron shareholder.
